# MED25 Is a Mediator Component of HNF4α-Driven Transcription Leading to Insulin Secretion in Pancreatic Beta-Cells

**DOI:** 10.1371/journal.pone.0044007

**Published:** 2012-08-30

**Authors:** Eun Hee Han, Geun Bae Rha, Young-In Chi

**Affiliations:** Section of Structural Biology, Hormel Institute, University of Minnesota, Austin, Minnesota, United States of America; University of British Columbia, Canada

## Abstract

Unique nuclear receptor Hepatocyte Nuclear Factor 4α (HNF4α) is an essential transcriptional regulator for early development and proper function of pancreatic ß-cells, and its mutations are monogenic causes of a dominant inherited form of diabetes referred to as Maturity Onset Diabetes of the Young 1 (MODY1). As a gene-specific transcription factor, HNF4α exerts its function through various molecular interactions, but its protein recruiting network has not been fully characterized. Here we report the identification of MED25 as one of the HNF4α binding partners in pancreatic ß-cells leading to insulin secretion which is impaired in MODY patients. MED25 is one of the subunits of the Mediator complex that is required for induction of RNA polymerase II transcription by various transcription factors including nuclear receptors. This HNF4α-MED25 interaction was initially identified by a yeast-two-hybrid method, confirmed by *in vivo* and *in vitro* analyses, and proven to be mediated through the MED25-L*XX*LL motif in a ligand-independent manner. Reporter-gene based transcription assays and siRNA/shRNA-based gene silencing approaches revealed that this interaction is crucial for full activation of HNF4α-mediated transcription, especially expression of target genes implicated in glucose-stimulated insulin secretion. Selected MODY mutations at the L*XX*LL motif binding pocket disrupt these interactions and cause impaired insulin secretion through a ‘loss-of-function’ mechanism.

## Introduction

Hepatocyte Nuclear Factor 4α (HNF4α) is a unique member of the nuclear receptor (NR) superfamily, and plays a critical role in early vertebrate development and metabolic regulation [1]. It is highly expressed in the liver, kidney, intestine and pancreas, and its crucial role in these vital organs has been proven by a recent genome-wide expression profiling study [2] and conditional inactivation of its gene in mice [3], [4], [5]. HNF4α regulates expression of a wide variety of essential genes, including those involved in liver and pancreatic cell differentiation, embryogenesis and early development, glucose metabolism, lipid homeostasis, and amino acid metabolism. As such, mutations in *HNF4α* cause a dominantly inherited form of diabetes known as Maturity Onset Diabetes of the Young 1 (MODY1) [6], further underscoring its pivotal role in human pancreatic ß-cell function and metabolic regulation [4], [7].

As a member of the NR superfamily, HNF4α is comprised of distinctive modular domains and exerts its function through various molecular interactions via combinatorial recruitment of multi-protein complexes, including transcriptional cofactors and mediators that further regulate the remodeling of chromatin structure at target gene promoters through histone modifications [8]. Well-known transcriptional coregulators of HNF4α include p160/SRC coactivators such as SRC1 and GRIP1 [9], NR corepressors such as NCoR and SMRT [10], CBP [11], and PGC-1α [12]. However, its entire protein recruiting network is not well characterized, and more key regulators likely remain to be discovered. Thus, in order to identify additional functional binding partners of HNF4α working in ß-cells, we performed yeast-two-hybrid experiments using various constructs of HNF4α as bait and a ß-cell library as prey, and identified MED25/TRAP97 (also known as DRIP97, ARC92, p78, ACID1, or PTOV2) as the Mediator component of HNF4α-driven transcription.

Mediator is an evolutionally conserved multi-subunit coactivator complex that transmits gene regulatory signals by serving as a molecular bridge between transcription factors (as well as associated transcriptional coactivators) to the general transcription machinery containing RNA polymerase II [13], [14]. Mediator does not possess intrinsic histone/chromatin-modifying activities and is commonly associated with other general coactivators that contain chromatin-modifying/remodeling activities such as p160/SRC coactivators or CBP. In humans, the Mediator complex was first identified as a positive regulator of thyroid hormone receptor (TR)-mediated transcription and characterized as a TR-associated protein (TRAP) complex [15]. Mediator has since been recognized as a broadly functional coactivator for NRs and other gene-specific transcription factors [16], [17]. The mammalian Mediator complex is composed of at least 28–30 subunits of varying sizes and compositions [13], [17] which display a dynamic nature of association and dissociation. These subunits are organized as a tightly associated core sub-complex, and associate with several groups of subunits that are believed to constitute distinct modules. Although the complex seems to be universally required for all genes, specific subunits are dedicated to regulation of distinct expression programs via interactions with relevant gene-specific transcriptional activators.

In this work, we present evidence that MED25 is recruited by HNF4α in a ligand-independent manner, and this interaction is necessary for full HNF4α-mediated transcription leading to insulin secretion in the pancreatic ß-cells. This interaction is disrupted by the two MODY mutations at the L*XX*LL motif binding pocket, and normal insulin secretion is impaired as a result. These observations suggest a key role for MED25 as an active Mediator component in HNF4α signaling, which can be exploited for more effective therapeutic intervention.

## Materials and Methods

### Yeast Two-hybrid

The initial screening using the lexA system was carried out through the Yeast Model System Genomics (YMSG) facility at the Duke University. HNF4α-ligand binding domain (LBD) (142–368) was cloned into the bait vector pGBKT7short (a modified version of pGBKT7 (Clontech) with tags between Gal4BD and bait removed) and the mouse pancreatic library (Clontech) was used as prey. Self-activation was tested with an empty Gal4BD bait vector before the screening against the library. Two hybrid screens were performed with a standard method using the yeast strain *S. cerevisiae* AH109. Primary isolates were re-streaked on trp−/leu−/his−/3 mM 3-AT plates and grown several days for lacZ assays. Positive colonies that showed a color change in LacZ assays were picked for colony PCR or for isolation of DNA. Approximately 6×10^6^ independent transformations were screened, of which 27 clones were positive for lacZ assays. Each positive hit was retested for one to one interaction by examining the growth of the transformant and performing lacZ assays. Subsequently, the positive clones were sequenced using automated DNA sequence analysis (ABI) and homologies were identified using BLASTN/BLASTX (National Center for Biotechnology Information).

### Construction of expression vectors

The luciferase reporter plasmids, pCMV Sport6 MED25 harboring the full length cDNA of Human MED25 (or PGC-1α), pcDNA3 HNF4α FL containing the full length of human HNF4α, and firefly reporter vector pGL3 (BA1)_3_ were constructed as described previously [18]. The same vectors were used for transfection and insulin secretion assays. For *in vitro* binding studies, recombinant HNF4α-LBD proteins with a TEV cleavage site were cloned into pET41a (Novagen) with a GST tag, while the full-length PGC-1α or MED25 proteins containing the L*XX*LL motif (wt or the NR mutant) were also cloned into pET41a (Novagen) vector.

### MED25 NR and HNF4α MODY Mutant Generation

The ‘Quick Change Multi Site-Directed Mutagenesis’ kit (Stratagene) was used to generate the MED25-NR (L*XX*LL) and HNF4α MODY mutant constructs. The plasmid templates used in the mutagenesis protocol were pCMV Sport6 MED25 and pcDNA3 HNF4α-FL. All of the generated constructs with the mutated sequences were verified with DNA sequencing.

### Cell Culture

For transcriptional assays, HeLa cells were cultured in DMEM medium supplemented with 10% fetal bovine serum, 50 units/ml penicillin G, 50 µg/ml streptomycin (Sigma), and 0.1 mM non-essential amino acids (Invitrogen). For the remaining cell culture experiments, mouse insulinoma 6 (MIN6) cells of passage 28 through 30 were cultured in Dulbecco’s modified Eagle’s media (DMEM) containing 5 mM glucose, 10% (vol/vol) fetal bovine serum (FBS), 1% penicillin/streptomycin, 2 mM glutamine, and 100 µM β-mercaptoethanol [19]. MIN6 cells were a kind gift from Dr. Sabire Özcan at the University of Kentucky [20].

### Transient Transfection and Transcription Assays (Luciferase Reporter Assays)

The full length cDNA of human HNF4α wt or the mutant were subcloned into the pcDNA3(+)/Neo vector (Invitrogen), and the reporter vector pGL3-(BA)_3_ containing three copies of the HNF4α response element within the promoter of human *Apolipoprotein B* was constructed and used for luciferase assays in the absence or presence of transfected coactivators (MED25 or PGC-1α). HeLa cells were transfected using Opti-MEM and LipofectAMINE 2000 reagent (Invitrogen) according to the manufacturer’s recommendations. Briefly, a total of 30 ng of pcDNA3 HNF4α and 150 ng of pCMV Sport6 PGC-1α, 50 ng of pCMV Sport6 MED25, 50 ng of pGL3 (HNF1α)_1_ and 10 ng of pRL-TK (control renilla luciferase vector) were used for transfection of 1×10^5^ cells seeded on 24-well plate one day before transfection. For RNA interference experiments, 20 pmol of siRNA of MED25, HNF4α, and PGC-1α were used for inhibition. For ERα and PPARγ luciferase assays, 6 hrs after transfection, cells were treated with 10 nM of estradiol or 1 µM of Troglitazone for 48 hrs. These additional luciferase vectors were kind gifts from Dr. Dan Noonan. After transfection and incubation, cells were washed with 1× PBS and lysed with luciferase lysis buffer supplied with the Luciferase assay kit (Promega). Luciferase activity was measured using the Dual Luciferase assay system (Promega) and Lmax Luminometer (Molecular Devices). All values were normalized by the relative ratio of firefly luciferase activity and renilla luciferase activity. At least four independent transfections were performed in duplicate.

### Glutathione S-transferase (GST) Pull-down Assays

HNF4α-LBD GST fusion proteins were produced in *Escherichia coli,* while [^35^S]-labeled MED25 (wt and the NR mutant), and PGC-1α wt were produced with a TNT reticulocyte lysate *in vitro* transcription and translation kit (Promega). About 1 µg of fusion proteins bound on beads were resuspended in 50 µl of GST pull down buffer (50 mM Tris pH 8.0, 100 mM NaCl, 0.5 mM EDTA, 0.1% NP-40), and mixed with 50 µl of the *in vitro* translate before being allowed for binding at 4°C for 4 hrs. Immobilized GST fusion protein was detected by SDS-PAGE and Western blotting using Coomassie blue staining and probing with a GST specific antibody (GE Healthcare). The beads were then washed 3 times with 1 ml of GST pull down buffer and resuspended in SDS–PAGE sample buffer. After electrophoresis, the [^35^S]-labeled proteins were detected by autoradiography.

### Co-immunoprecipitation

MIN6 cells were transfected with pCMV Sport6 MED25 and pcDNA3 HNF4α using Metafectene pro reagent (Biontex). 48 hours after transfection, cells were washed with 1× PBS and lysed with 1 ml of IP lysis buffer (50 mM Tris pH 7.4, 150 mM NaCl, 1 mM EDTA, 0.5% NP-40, 5% glycerol) supplemented with a protease inhibitor cocktail mix (Roche). Cell lysates were pre-cleared by pre-incubation with protein A Sepharose 4 Fast Flow beads (GE Healthcare) for 15 min, and incubated for 2 hrs with the beads and a 1∶200 dilution of HNF4α and anti-rabbit IgG antibodies. The beads were then washed once with IP lysis buffer and twice with PBS, and the immune complexes were released from the beads by boiling in sample buffer for 5 min. Following electrophoresis on 10% SDS-PAGE, immunoprecipitates were transferred onto PVDF membrane, and immunoblotted with a specific MED25 antibody (Abgent). Proteins were visualized using the enhanced chemiluminescence (ECL) detection system (GE Healthcare).

### Western Blotting

Immunodetection of expression of MED 25 in MIN6 cells was performed using whole cell lysates, rabbit anti- MED 25 antibody (Abgent), mouse anti-Actin antibody (Santa Cruz), and goat anti-rabbit antibody conjugated with HRP (Cell Signalling Technology). Briefly, transfected cells were lysed with cell lysis buffer (20 mM Tris pH 7.5, 150 mM NaCl, 0.5% TritonX-100, 0.5% NP-40, and 1 mM EDTA) with protease inhibitors. Cell lysates were centrifuged and the protein concentration of each cell lysate was determined using the Bio-Rad protein assay reagent. The same amounts of cell lysates were loaded onto a 10% SDS-PAGE, and the proteins were fractionated by electrophoresis and transferred to a PVDF membrane. The blotted membrane was incubated with aforementioned antibodies and signals were detected using the ECL-based method (GE Healthcare).

### Gene Knock-down (siRNA or shRNA) for Cellular Studies

The sequence of MED25-siRNA used for initial transcription assays was described elsewhere [21]. A negative control scrambled siRNA (Ambion) was used to demonstrate that transfection did not induce nonspecific effects on gene expression. One microgram of each siRNA oligo for every 1×10^6^ cells was electroporated into HeLa cells using the Amaxa nucleofactor and cell line nucleofactor kit V according to manufacturer’s protocols. Cells were then incubated in DMEM containing 5 mM glucose and 10% FBS. After 12 hr incubation, cells were switched to DMEM containing 5 mM glucose, 10% FBS, and antibiotic/antimycotic solution for an additional 36–40 hrs. Cells were then processed for luciferase assays.

We also used short hairpin RNA (shRNA) for MED25 interference in the later real time PCR/Q-PCR and insulin secretion assays. MED25-shRNA was purchased from Origene with the following sequences: 5′-GAC CAG AGC GGC TTC GTC AAT GGC ATC CG-3′. Cells grown to 50% confluence were transfected using Metafectene Pro Transfection reagent (Biontex) with MED25 or scrambled shRNA according to the manufacturer’s instructions.

### Quantitative Real Time PCR

MIN6 cells were transfected with MED25 (wt or the NR mutant), HNF4α (wt or the MODY mutant) plasmids. 24 hours after transfection, total RNA was extracted using Tri reagent (Sigma Aldrich) and 0.5 µg of RNA was reverse transcribed using Onestep RT-PCR kit (Qiagen) and amplified by PCR whose product formation was monitored continuously during PCR using Sequence Detection System software (ver. 1.7; Applied Biosystems). Accumulated PCR products were detected directly by monitoring the increase of the reporter dye (SYBR). The expression levels of PPARα, L-pyruvate kinase (PK), Kir6.2, and GLUT2 in the exposed cells were compared to those in control cells at each time point using the comparative cycle threshold (Ct)-method. The quantity of each transcript was calculated as described in the instrument manual and normalized to the amount of actin, a housekeeping gene.

### Semi-Quantitative Polymerase Chain Reaction (Q-PCR)

MIN6 cells were transiently transfected with MED25 (wt or the NR mutant) and/or HNF4α (wt or the MODY mutant) and MED25 shRNA using Metafectene pro transfection reagent (Biontex) for 24 hrs. Total RNA from the treated cells was prepared with the Tri reagent (Sigma Aldrich) according to the manufacturer’s protocol. cDNA synthesis and semi-quantitative PCR for PPARα, L-PK, Kir6.2, and GLUT2 mRNA were performed, and the PCR reactions were electrophoresed using a 2% agarose gel. The band intensities of the amplified DNA products were visualized using the SYBR Green I DNA gel stain kit (Invitrogen).

### Insulin Secretion Assays

This functional study was performed with the MIN6 cell line which exhibits the characteristics of glucose metabolism and glucose-stimulated insulin secretion similar to those of normal islets [22]. To quantify the amount of insulin secreted, MIN6 cells were grown on a 6-well dish (about 1×10^6^ cells) and transfected with various vectors harboring MED25, HNF4α, MED25 NR mutant, MED25 shRNA, or HNF4α MODY mutants and incubated for 24 hrs. After the incubation period, the cells were washed three times with KRB buffer (119 mM NaCl, 4.7 mM KCl, 2.5 mM CaCl2, 1.2 mM MgSO4, 1.2 mM KH2PO4, 25 mM NaHCO3, 10 mM Hepes pH 7.4, and 0.1 g BSA), and further incubated in 1 ml pre-warmed KRB buffer containing 1 mM glucose for 1 hr at 37°C. The cell culture media (total of 1 ml) was collected and used to measure the level of insulin release with the insulin ELISA kit (Mercodia) by means of an enzyme immunoassay followed by an optical density reading at 450 nm. The amount of stimulation (fold increase) refers to insulin secretion after various treatments relative to insulin secretion in 1 mM glucose treated cells, which was set as 1-fold. Values are expressed as means ± SD of data obtained from three independent experiments (n = 3), each performed in duplicate.

### Statistical Analysis

Presented data are expressed as mean ± standard error of the mean (SEM) of at least three independent groups. Statistical significance was determined by one-way ANOVA followed by Student-Newman-Keuls method using Sigma Stat 3.1 software (Systat Software, San Jose, CA). A probability value p<0.05 was considered statistically significant.

## Results

### Initial Identification of HNF4α/MED25 Molecular Interactions

**Figure 1:**
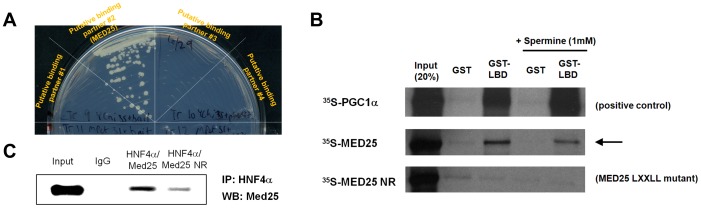
A single subunit, MED25, binds directly to HNF4α in a ligand-independent manner. (A) Yeast-two-hybrid interaction of MED25 and HNF4α-LBD (positive colony in the sector at the 11 O’Clock direction) shown together with negative interacting candidates. One-to-one co-transformed yeasts were selected for growth on Trp−/Leu- plates, and streaked onto Trp−/Leu−/His−/3 mM 3-AT plates. (B) GST pull-down evidence of the interactions. *In vitro* translated and ^35^S labeled MED25 (wt or the L*XX*LL mutant) was incubated with immobilized GST-tagged HNF4α-LBD in the absence of any external ligand. PGC-1α was used as a positive control while GST alone was used as a negative control (lanes #2 and 4). The MED25 L*XX*LL mutant (MED25 NR) failed to interact, and the addition of 1 mM spermine did not affect the interactions. (C) Immunoblotting evidence of the interactions. Nuclear extracts of MIN6 cells transfected with HNF4α and MED25 were subjected to immunoprecipitation with either IgG (negative control) or HNF4α antibodies followed by Western blot of the immunoprecipitates with MED25 subunit antibodies (input amount on the left).

The current model of eukaryotic gene regulation is best described by the combinatorial recruitment involving multiple transcriptional regulators [23], [24]; however, the full extent of tissue-specific and protein-specific recruitment has not been well characterized. Thus, to elucidate the detailed molecular interaction network of HNF4α-mediated transactivation in pancreatic ß-cells, we performed a yeast two-hybrid screen and identified MED25 as one of the binding partners of HNF4α. The bait vectors containing various constructs of HNF4α were constructed and screened against the pre-transformed mouse pancreatic library as prey. MED25, among others, including the well-known nuclear receptor coactivator 2 (NCoA2) [25], was identified as a putative binding partner when the HNF4α-ligand binding domain (LBD) was used as bait, and was positively retested for one to one interaction through examining the growth of the transformant (Figure 1A). DNA sequence analysis of the positive clones revealed three cDNAs derived from the same gene, encompassing the 407–744 region of MED25 (AAH21333) containing the LXXLL motif. This mouse sequence shares 91% identity with its human counterpart.

### Physical Interaction between HNF4α and MED25

To confirm their physical interactions, GST pull-down assays were carried out using a GST fusion protein of HNF4α-LBD and *in vitro*-translated MED25 along with PGC-1α as positive control. As shown in Figure 1B, [^35^S]-labeled MED25 wt showed evidence for interaction with the HNF4α-LBD in the absence of additional external ligand (although much weaker than HNF4α/PGC-1α interactions) while the MED25 mutant (MED25 NR) in which the sole L*XX*LL sequence (646–650) was mutated to L*XX*AA failed to interact, suggesting its involvement in this interaction. The previous studies with MED1 and NRs noted that a polyamine such as spermine significantly enhances the binding [26]; however, no such effect was observed for the interaction between HNF4α and MED25 (Figure 1B).

To further confirm their interactions *in vivo*, co-immunoprecipitation experiments were carried out with whole-cell extracts derived from MIN6 (mouse insulinoma 6) cells. Full-length HNF4α and MED25 (or MED25 NR) were overexpressed, and the cell lysates were subject to immunoprecipitation with HNF4α polyclonal antibody and control IgG antibody before running on a 10% SDS-PAGE gel and performing Western blot analysis with MED25 antibody. As shown in Figure 1C, MED25 was efficiently coprecipitated in MIN6 cells with HNF4α and MED25 NR was only weakly coprecipitated while the control IgG was not. The weak *in vivo* residual interaction by MED25 NR suggests a possible involvement of other regions of MED25 for HNF4α interactions although the L*XX*LL motif plays a major role. Taken together, these binding analysis results prove that MED25 physically interacts with HNF4α in living cells and present MED25 as a potential candidate for anchoring the Mediator complex to HNF4α-responsive promoters.

### MED25 Mediates HNF4α Transactivation and MED25 Involvement is Specific to a Selective Set of Nuclear Receptors

**Figure 2:**
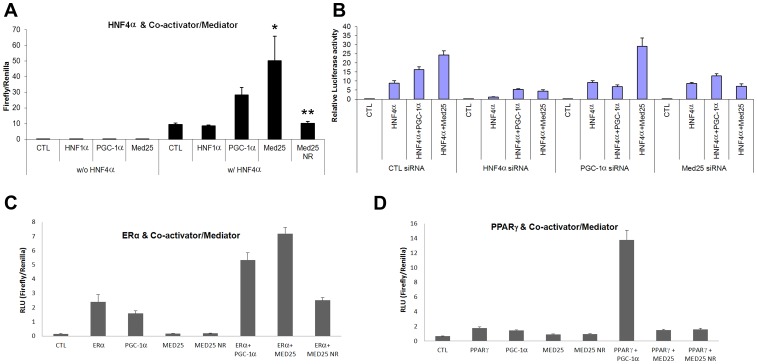
Effects of MED25 on HNF4α and other NR-mediated transcription. (A) Overall transcriptional activity measured by standard luciferase-based transcriptional reporter assays on a HNF4α-responsive element. The left four lanes are without HNF4α transfection (thus single transfection of the indicated protein) and the right five lanes correspond to the ones with HNF4α transfection (thus double transfections of HNF4α- and the respective protein expressing vectors). HNF4α serves as a negative control, while PGC-1α serves as a positive control. MED25 NR refers to the LXXLL mutant. CTL in the first lane of each half refers to an empty vector, and all data have been normalized against firefly *Renilla* luciferase activity. Data represent the mean±SE (n = 4). Asterisk (*) indicates a significant difference between MED25 plus HNF4α and control (CTL) while (**) indicates a significant difference between MED25 plus HNF4α and MED25 NR plus HNF4α. (B) Gene knockdown experiments to test the specific protein effects. Luciferase assays for HNF4α-mediated transcription after transfections with the indicated proteins and the respective siRNA. CTL siRNA refers to scrambled RNA as a negative control while PGC-1α serves as a comparative positive control. (C) Effects of MED25 on ERα-mediated transcription. Luciferase assays of single transfection or double transfections with the indicated proteins in the presence of 10 nM Estradiol, a potent ligand for ERα. MED25 has a strong impact on ERα-mediated transactivation. (D) The same set of experiments as (C) for PPARγ in the presence of 1 µM Troglitazone, an agonist for PPARγ. MED25 shows no significant influence on PPARγ-mediated transactivation.

Since MED25 is a component of the eukaryotic transcriptional Mediator complex, we first tested its involvement in HNF4α-mediated transcription by over-expressing both proteins and measuring the changes in the reporter gene expression level by HNF4α luciferase assays. As shown in Figure 2A, MED25 substantially enhanced the expression of the reporter gene while two other controls (empty vector (CTL) and another MODY gene product HNF1α) showed no increase. The increase in transcription promoted by the addition of MED25 was even greater than that by the addition of PGC-1α, despite its weaker *in vitro* binding (Figure 1B), implying that strong *in vitro* interactions between HNF4α and its transcriptional regulators do not necessarily reflect functionality on endogenous promoters. This may also imply that the Mediator complex plays a more dominant role in overall transactivation than coactivators [27], although we cannot rule out the possibility that these weaker transcription factor-Mediator interactions are further supported by additional protein-protein interactions *in vivo*. This involvement of MED25 in HNF4α-mediated transcription was substantially attenuated by mutations in the L*XX*LL motif (MED25 NR), indicating that this activation is MED25-specific and once again the C-terminal L*XX*LL motif in MED25 is important for this interaction.

The involvement of MED25 in HNF4α-mediated transcription was next tested by knockdown of individual proteins using siRNA prior to forced expression of proteins of interest including PGC-1α as a control (Figure 2B). Knockdown of each protein (HNF4α, PGC-1α, or MED25) followed by overexpression of HNF4α alone or HNF4α with a coactivator/Mediator partner resulted in knockdown-specific reduction of HNF4α-mediated gene expression in the reporter assays, suggesting the direct involvement of MED25 in HNF4α-mediated transactivation.

In order to investigate whether MED25 is also recruited by other NRs, a selective set of NRs have been tested by similar overexpression and transcription assays. We chose PPARγ, ERα, PR, RARα, and RXR for the studies, among which only ERα showed a positive response to the overexpression of MED25 (Figure 2C). Other NRs showed no significant responses (Figure 2D and Figure S1), while they all showed positive response to the overexpression of PGC-1α. These results indicate that PGC-1α is recruited by many NRs as a general coactivator, while a specific subunit of the Mediator complex (or sometimes more than one) is recruited by each NR [13], [20] including MED25 as a specific Mediator component for HNF4α and ERα. Like the MED25-HNF4α interactions, the positive response on ERα-mediated transcription by MED25 was attenuated by the overexpression of the MED25 NR mutant (Figure 2C), again suggesting the involvement of the L*XX*LL motif in this interaction and activation.

### MED25 Enhances HNF4α Target Gene Expression Leading to Glucose-stimulated Insulin Secretion in ß-cells

MODY patients are mainly characterized by a severe impairment of insulin secretion [28], [29], and MODY gene products, including HNF4α, are monogenic causes of an insulin secretion defect resulting in diabetes. To probe the involvement of MED25 in HNF4α subtype specific target gene expression and insulin secretion in ß-cells, we next tested whether MED25 is required for HNF4α transcriptional activation of previously known HNF4α target genes directly involved in ß-cell insulin secretion such as PPARα [3], L- pyruvate kinase (L-PK) [30], [31], GLUT2 [30], [31], and Kir6.2 [3], [4]. These proteins are involved in the insulin secretion signalling pathway at certain stages such as glucose sensing and transport (GLUT2), TCA or Krebs cycle, i.e. ATP production by mitochondrial enzymes (L-PK), ATP-dependent potassium channel (Kir6.2), and transcriptional regulation of additional gene products along the pathway (PPARα).

**Figure 3:**
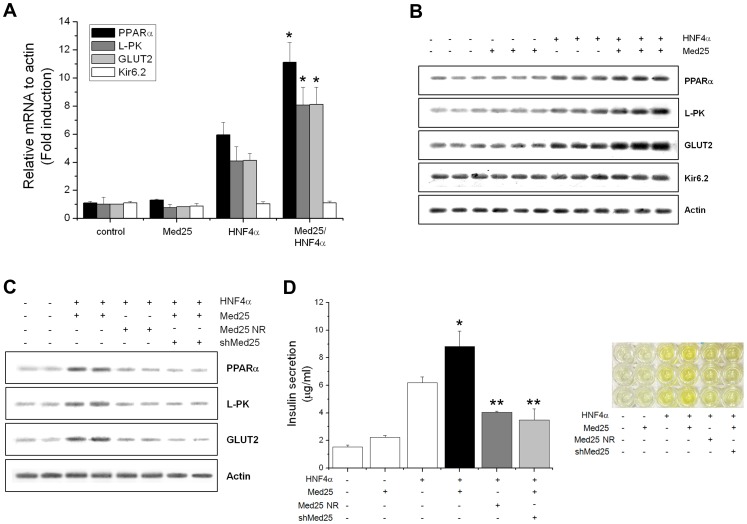
Effects of MED25 on HNF4α target gene expression implicated in insulin secretion from pancreatic ß-cells. (A) The RNA amounts were quantified by real-time PCR with the primers against each HNF4α target genes following transfection of the indicated expression vectors. Control indicates transfection with an empty vector, and error bars indicate the range of experiments performed in duplicate. Each graph bar corresponds to each target gene as indicated in the inset box. PPARα displayed the highest response (12-fold increase) followed by L-PK and GLU2 (both 8-fold increases), while Kir6.2 showed no response. (B) Q-PCR results of the same RNA quantifications. The experimental results are shown in triplicate while actin, a house-keeping gene product, was used as a loading control in the bottom panel. These results are in good agreement with the real time PCR data shown in (A). Data represent the mean±SE (n = 3). Compared with HNF4α alone, *p<0.01. (C) Effects of the MED25 NR mutant and shMED25 on HNF4α target gene expression shown by Q-PCR. The experimental results are shown in duplicate and again actin was used as loading control. They all showed the MED25-specific effects. (D) Effects of the MED25 NR mutant and shMED25 on insulin secretion shown by color change (right) and its quantification by absorption at 450 nm (left). Darker colors indicate more insulin secretion as shown by higher bars in their actual numerical value plots. These data show the direct correlation between HNF4α target gene expression and insulin secretion. Data represent the mean±SE (n = 3). Compared with the control, *p<0.01. Compared with MED25 plus HNF4α, **p<0.01.

Target gene expression levels were measured by means of transient transfection in MIN6 cells followed by quantification of enriched DNA by real time PCR and Q-PCR. Our results showed that MED25 was necessary for full activation of the majority of HNF4α subtype specific target genes involved in insulin secretion. As shown in Figures 3A and B, expressions of aforementioned HNF4α target genes were mostly increased upon transfections of HNF4α and MED25. PPARα displayed the highest response to double transfection (12-fold increase) followed by L-PK and GLUT2 (both 8-fold increases), while Kir6.2 showed no response. Although the ATP-dependent potassium channel is one of the central players in glucose-stimulated insulin secretion, and altered potassium channel activity is related to the impaired insulin secretion in MODY patients [32], [33], there are contradicting data on whether or not one of its subunits, Kir6.2, is a direct target of HNF4α. While one group reported that Kir6.2 is a target gene of HNF4α in ß-cells through conditional HNF4α knockout experiments [4], another group reported that the expression level of Kir6.2 in HNF4α knockout mice was unchanged as compared with control mice [3]. Our findings partially support the latter observation, although we cannot rule out the possibility that MED25 involvement is for only a subset of endogenous HNF4α target genes and another Mediator subunit might be involved in HNF4α-mediated Kir6.2 transactivation.

The involvement of MED25 in HNF4α-mediated transcription and insulin secretion was further tested using the MED25 LXXLL-motif mutant (MED25 NR) and the knockdown of MED25 using shRNA (Figure S2 for validation of MED25 knockdown by shRNA). In keeping with the results from siRNA treatments for the luciferase assays, the expression of all three target genes in ß-cells (PPARα, L-PK, and GLUT2) were markedly reduced by both the MED25 NR mutant and MED25 shRNA treatment (Figure 3C). As a result, the overall insulin secretion from MIN6 cells was also reduced significantly by both treatments (Figure 3D). Taken together, these preliminary data indicate that MED25 is a critical Mediator component involved in HNF4α-mediated transcription that promotes glucose-stimulated insulin secretion in pancreatic ß-cells.

### MED25 Lacks Synergistic Activation with MED1, Another Mediator Component Known for HNF4α

**Figure 4:**
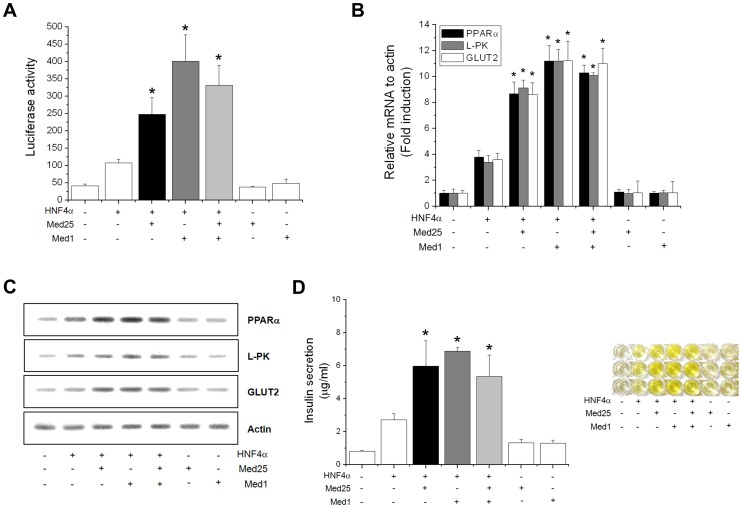
Lack of synergistic activation by MED25 and MED1 on HNF4α target genes and insulin secretion. (A) Overall transcriptional activity measured by standard luciferase-based transcriptional reporter assays on a HNF4α-responsive element. Data represent the mean±SE (n = 4). Compared with HNF4α alone, *p<0.01. (B) The RNA amounts were quantified by real-time PCR with the primers against each HNF4α target genes following transfection of the indicated expression vectors. Data represent the mean±SE (n = 3). Compared with HNF4α alone, *p<0.01. (C) Q-PCR results of the same RNA quantifications showing similar effects. (D) Effects of both MED25 and MED1 on insulin secretion. These data indicate that each Mediator component individually act on HNF4α, and lack synergistic activation. Data represent the mean±SE (n = 3). Compared with HNF4α alone, *p<0.01.

MED1, the best-studied Mediator subunit, is also known to be involved in activation of HNF4α-mediated transcription [26], [34]. Thus, we tested for a potential synergistic activation by MED25 and MED1 towards HNF4α by co-transfection of both protein-producing vectors. As shown in Fig. 4, they appear to act individually on HNF4α, and lack synergistic activation. These effects are quite consistent throughout the transcription assays, target gene expression, and insulin secretion assays (Fig. 4A–D), indicating the linear relationship between the HNF4α target gene expression and insulin secretion in ß-cells.

It is interesting to point out that the effects by double transfection are intermediate values of the two individual transfections, possibly reflecting the fact that the functional unit of HNF4α is a dimer and each monomer has a roughly equal access to either MED25 or MED1, resulting in mixed complex formation and intermediate transactivation values. Since the L*XX*LL motifs of both MED25 and MED1 are essential for the physical interaction with HNF4α, both Mediator subunits are likely to compete for the same binding site on HNF4α and disallow synergistic activation. This lack of synergistic activation is consistent with the findings on MED14 and MED1 towards Glucocorticoid Receptor-mediated transcription [35], and MED25 and MED1 towards Retinoid Receptor-mediated transcription [21], although it is quite contrary to other Mediator subunit combinations such as MED19/MED26 or MED16/MED23 towards non-NR transcription factors [36], [37]. For NR activation, each Mediator components appear to act independently and display distinct protein recruitment patterns [21], [35] although we cannot rule out the possible involvement of additional Mediator components through L*XX*LL motif-independent interactions.

### Disruptive Effects by MODY Mutations Near the L*XX*LL Motif Binding Pocket

**Figure 5:**
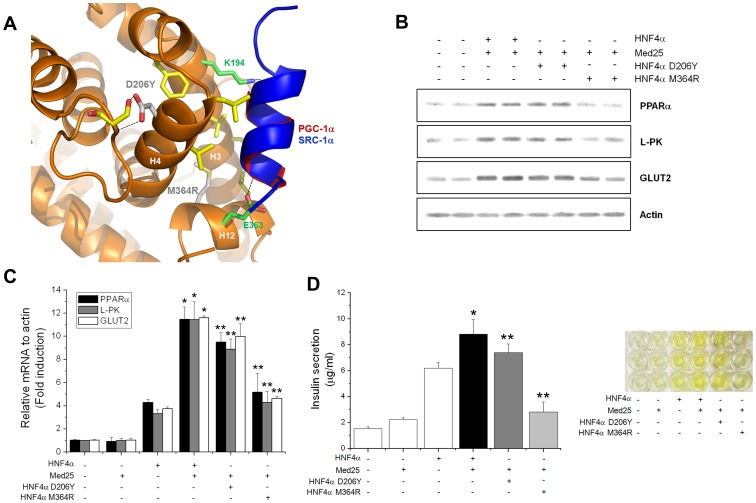
MODY mutational effects on HNF4α-MED25-mediated transactivation and insulin secretion. (A) Close-up view of the L*XX*LL motif-mediated coactivator binding pocket of HNF4α, showing the locations of the selected MODY mutations for the current studies (D206Y and M364R) highlighted by a ball-and-stick model (gray sticks and labels). The bound SRC-1α L*XX*LL motif peptide (blue) and PGC-1α L*XX*LL motif fragment (red) from the known crystal structures (PDB accession codes 1PZL and 3FS1, respectively) are shown as ribbon diagrams. Hydrogen bonds are indicated as dashed lines. The hydrophobic residues lining the L*XX*LL motif binding groove and the ‘charge-clamp’ residues (K194 and E363) that make hydrogen bonds with the backbone atoms of the bound peptides are also highlighted as yellow and green sticks, respectively. (B) Mutational effects on HNF4α target gene expression implicated in insulin secretion. RNA amounts were quantified by real-time PCR with the primers against each HNF4α target gene. The first set of bars indicates the control experiments where no transfection vectors were added. Each target gene expression is similarly affected by the mutations, and the mutational effects are more pronounced for the M364R mutant in all cases (last set of bars). (C) Q-PCR results of the same mRNA quantification showing similar effects. Data represent the mean±SE (n = 3). Compared with HNF4α alone, *p<0.01. Compared with MED25 wt plus HNF4α, **p<0.01. (D) Effect of MODY mutations on insulin secretion as shown in Figure 3D and 4D. Similar mutational effects are observed, indicating a direct functional linkage between target gene expression and insulin secretion. Data represent the mean±SE (n = 3). Compared with HNF4α alone, *p<0.01. Compared with MED25 wt plus HNF4α, **p<0.01.

Several mutations have been identified from the MODY patients and point mutations can be very instructive site-specific indicators of protein function and structure. Thus, we probed the effects of the two MODY point mutations (D206Y and M364R) found near the L*XX*LL motif binding site for MED25 recruitment, target gene expression and insulin secretion (Figure 5A).

The selected mutations are proximal to the L*XX*LL motif binding pocket, and any substitutions at these positions are believed to influence coactivator/Mediator recruitment. While the D206 residue is located on the first turn of helix H4 at the fringe of the L*XX*LL motif binding pocket, M364 is located at the rim of the L*XX*LL motif binding groove (Figure 5A). Previously, the mutational effects of these two MODY point mutations have been thoroughly investigated by us through a series of experiments on their structural integrity and specific functional roles such as coactivator recruitment, overall transcription, ligand selectivity, and target gene recognition [38]. For protein interaction assays, together with PGC-1α and SRC-1α, MED25 interactions were measured with HNF4α-LBD wt and the MODY mutants [38]. While the D206Y mutation has subtle effect, the M364R mutation significantly impaired MED25 interactions, suggesting that although the neighboring residues such as D206 also play a role, the actual makeup of the hydrophobic groove seem to play a more predominant role in L*XX*LL motif recognition and coactivator/Mediator recruitment [39], [40].

In agreement with the protein binding assays, insulin-secretion related target gene expression and the subsequent insulin secretion decreased in MODY mutants. As shown in Figures 5B and 5C, expression of each target gene is similarly affected by the mutations, and the mutational effects are more pronounced for the M364R mutant in all cases (last set of bars), as measured by both real time PCR and Q-PCR. Subsequently, insulin secretion was reduced to a similar extent (Figure 5D), implying that these gene products play critical roles in insulin secretion and that there is a linear relationship between the protein levels and insulin secretion. These functional disruptions are believed to be mainly due to their inability to recruit coactivators/Mediator complex to the fullest extent, while their DNA binding activities and ligand selectivities are preserved [38].

## Discussion

Transcription factors initiate transcription by recognizing their target genes and mediating additional interactions with various proteins including transcriptional coregulators and the Mediator complex to recruit the remainder of the main transcriptional machinery. To gain additional molecular insights into HNF4α function, we performed yeast two-hybrid screens and identified MED25 as a functional interacting partner of HNF4α in ß-cells.

Our study was focused on pancreatic ß-cells because HNF4α is one of the culprit gene products for a dominantly inherited form of diabetes, MODY, mainly characterized by the defect in insulin secretion from ß-cells. The interaction between HNF4α and MED25 was confirmed by *in vivo* coimmunoprecipitation and *in vitro* GST pull-down assays. The physiological implications of MED25-mediated transactivation of HNF4α was investigated by reporter gene assays and insulin secretion assays in combination with gene silencing studies. Recently, MED25 was identified as an associated subunit of Mediator with HNF4α also in the liver, in the context of lipid and detoxification gene expression [41].

The eukaryotic Mediator complex is made of multiple subunits and each transcription factor associates with a selective subunit for its transactivation [13], [20]. Several potential mechanisms for MED25-dependent Mediator recruitment to NRs have been suggested [21], [41]. In this report, we present further supporting evidence that MED25 is functionally recruited by several NRs such as HNF4α and ERα, indicating that MED25 is involved in gene selective activation of target genes by diverse NRs, but not all. Particularly, our findings establish MED25 as a potential anchoring point between the Mediator complex and the HNF4α necessary for full transactivation of HNF4α subtype specific target genes in ß-cells leading to insulin secretion. This interaction occurs in a ligand-independent manner, consistent with the current notion that HNF4α is a constitutively active NR [42], [43]. However, we failed to observe any synergistic activation between MED25 and MED1, another Mediator subunit known to be associated with HNF4α [26] towards HNF4α-mediated transcription. It appears that the synergistic activations between the Mediator subunits are absent in NR-mediated transcription as their L*XX*LL motifs are likely to compete for the same binding sites on NRs while the synergistic effects are more common in non-NR transcription factors [44].

Several mutations have been identified from MODY patients and previous studies on their mutational effects have shown that subtle disruptions of HNF4α’s molecular function can cause significant effects in afflicted MODY patients [38], [45]. Our findings confirm the structural predictions and prove that the principal molecular basis of their reduced transcriptional activities is their impaired ability to recruit the coactivators and the Mediator complex, thus establishing a direct linear linkage between the transcriptional events and the physiological consequences.

These results further prove that MODY mutations are loss-of-function mutations leading to impaired ß-cell function and demonstrate that the L*XX*LL motif interaction site, in addition to the more commonly exploited ligand binding pocket, can be an effective drug target site. NRs have been successful drug targets (about 20% of current pharmaceuticals) [46], [47], due to their modular structures and multiple key interaction sites (i.e. protein-ligand, protein-protein, and protein-DNA interactions). However, it has been believed that, for therapeutic intervention, complete inhibition or drastic modulation of these regulators is not desirable, and only partial agonists or antagonists should be considered [48], [49], [50]. These partial and selective modulators affect only a subset of functions or act in a cell-type-selective manner, and have substantial benefits by retaining the beneficial therapeutic effects while minimizing undesirable side effects. In order to improve the likelihood of finding desirable selective modulators, various potential target sites need to be explored and the NR-coactivator/Mediator binding pocket has been gaining much attention in recent years [51], [52]. We believe our newly obtained results will advance the current understanding of the molecular mechanisms underlying HNF4α function and suggest a better strategy for targeting this protein, especially its interaction with coactivators/Mediator complex, for therapeutic intervention [52], [53].

## Supporting Information

Figure S1 MED25 involvement in other NR-mediated transactivations (In Figure 2B, ER and PPARγ are shown as representative data. In this supplementary figure, additional data are shown for the remaining NRs): (A) progesterone receptor, PR, (B) retinoic acid receptor α, RARα, and (C) retinoid X receptor, RXR. They all showed negligible responses to MED25, while RARα and RXR showed strong responses to PGC-1α. PGC: PGC-1α, and NR: Med25 L*XX*LL mutant.
(TIF)

Figure S2 Knockdown efficiency of MED25 shRNA (shMed25) purchased from Origene tested along with scrambled RNA as a negative control. The protein levels in MIN6 cells were performed by specific MED25 antibody after transfections with MED25 and each shRNA (scrambled RNA or different amounts of shMed25) followed by Immunoblotting.
(TIF)
