# Supplementary figures and images for: MED25 Is a Mediator Component of HNF4α-Driven Transcription Leading to Insulin Secretion in Pancreatic Beta-Cells
Source: PLoS One. 2012 Aug 30;7(8):e44007. doi: 10.1371/journal.pone.0044007 (PMC3431373; doi:10.1371/journal.pone.0044007)

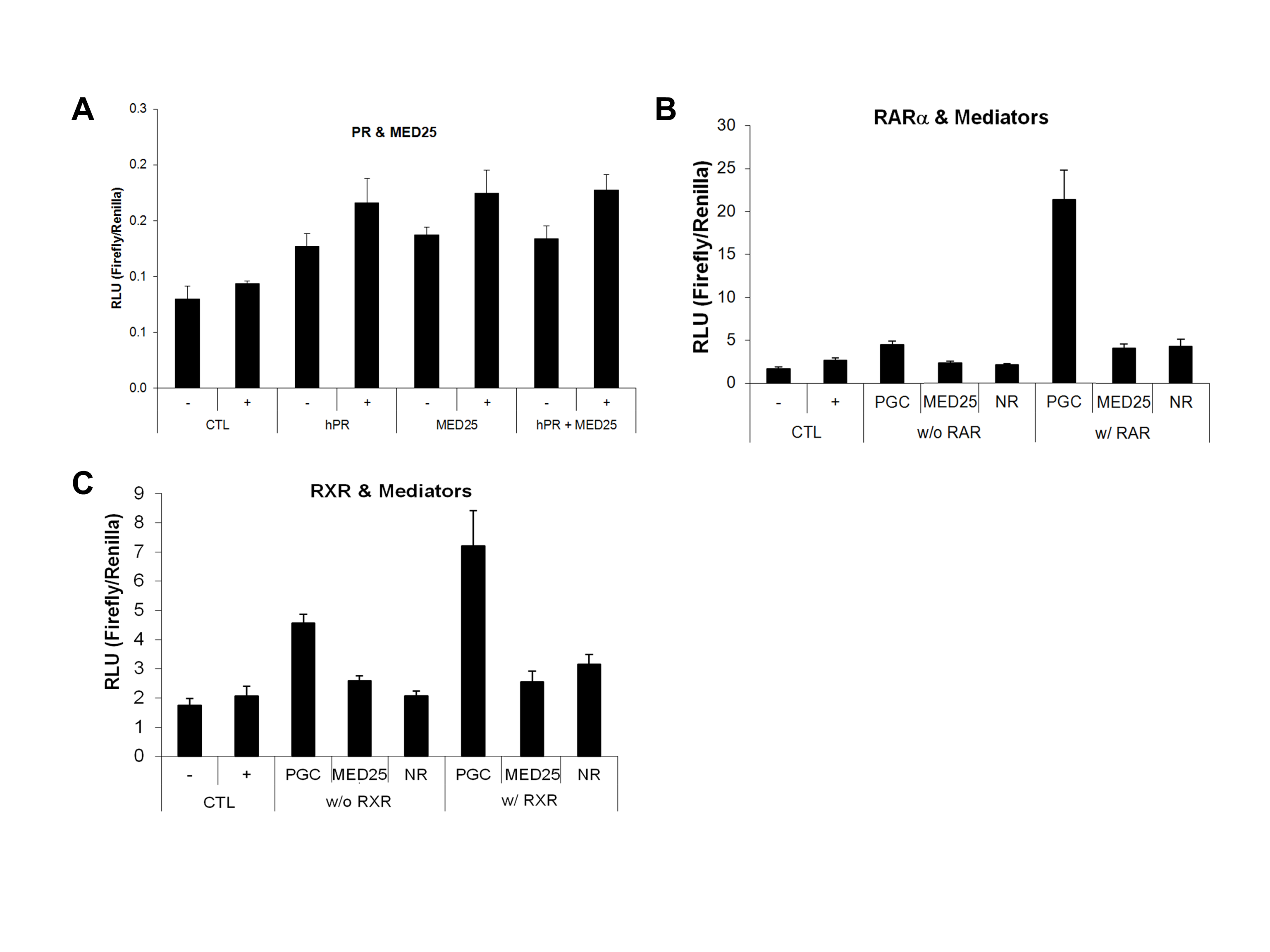

Supplement: Figure S1 — MED25 involvement in other NR-mediated transactivations (In Figure 2B, ER and PPARγ are shown as representative data. In this supplementary figure, additional data are shown for the remaining NRs): (A) progesterone receptor, PR, (B) retinoic acid receptor α, RARα, and (C) retinoid X receptor, RXR. They all showed negligible responses to MED25, while RARα and RXR showed strong responses to PGC-1α. PGC: PGC-1α, and NR: Med25 LXXLL mutant. (TIF) [file pone.0044007.s001.tif]
